# Malignant Hypertension Without End-Organ Damage Secondary to Stressful Condition in a Female

**DOI:** 10.7759/cureus.10109

**Published:** 2020-08-29

**Authors:** Hussain Hussain, Aya Fadel

**Affiliations:** 1 Internal Medicine, Cardiology Clinic, Pasadena, USA; 2 Internal Medicine, Florida International University, Hialeah Hospital, Miami, USA

**Keywords:** malignant hypertension, hypertensive emergency, physiological changes in stress, hypertension, catecholamines

## Abstract

Malignant hypertension (hypertensive emergency), is an extreme elevation of blood pressure under certain conditions that can lead to organ damage and other serious consequences. It is a common condition that affects about one in three Americans, according to the Centers for Disease Control and Prevention. An elevation of systolic blood pressure above 180 mmHg and diastolic blood pressure above 120 mmHg is considered a hypertensive emergency. This article addresses the case of a 61-year-old female patient who presented to the ER with a semicomatose and gasping condition and response to painful stimuli with an unclear voice. She also had unstable vital signs, with a blood pressure of 370/200 mmHg, a pulse rate of 115, a respiratory rate of 22, and a pulse oximetry of 96%, but no fever. Her son provided a brief history and reported that a stressful condition had occurred at home one hour before; she had begun to scream and been brought to the ER by ambulance in the condition described above. Cardiac monitoring and an electrocardiograph were performed and indicated a normal condition besides the unstable vital signs. Oxygen was administered via a nasal cannula with 20 mg of intravenous hydralazine, and the patient’s blood pressure improved progressively. Moreover, she regained consciousness with no end-organ damage. A hypertensive emergency is usually associated with end-organ damage, such as heart, kidney, eye, or brain damage. However, in this case, despite the extreme elevation of her blood pressure, the patient suffered no organ damage. It is essential to manage the extreme elevation of blood pressure as soon as possible and monitor the patient for consequences.

## Introduction

Hypertension is the most common condition in the United States. It affects one in three American adults, according to the Centers for Disease Control and Prevention [1]. Blood pressure is the pressure of the blood pushing against the arterial wall during its passage, and blood pressure varies depending on the activities performed. Hypertension increases the risk of heart disease, stroke, and other conditions. Systolic blood pressure measures arterial pressure during heartbeats, which normally should be less than 120 mmHg, whereas diastolic blood pressure measures arterial pressure between heartbeats or during heart rest, which should be less than 80 mmHg.

When blood pressure exceeds 120/80 mmHg, several readings reveal hypertension. Different levels of blood pressure occur: level one is considered normal, while level two indicates that a patient is at risk of prehypertension when his or her systolic blood pressure is between 120 mmHg and 129 mmHg and diastolic blood pressure is below 80 mmHg. Various evaluations consider blood pressure above 130/80 mmHg a hypertensive condition [2]. Hypertensive urgency occurs when blood pressure is equal to or above 180/110 mmHg with no organ damage and can be lowered safely within a few hours using medications. A hypertensive emergency entails elevated blood pressure equal to or above 180/110 mmHg and end-organ damage; mean arterial blood pressure should thus be lowered in the ICU over thirty minutes-one hour by up to 25% to prevent imminent organ damage [3].

## Case presentation

Clinical history

A 61-year-old female patient presented to the ER with a semicomatose and gasping condition. She was holding her right hand over the right side of her chest and responded only to painful stimuli with an unclear voice. Her son provided a brief history and reported that a stressful condition had occurred at home an hour before; she had been arguing with one of her family members for about 30 minutes, which had initiated her presentation. Her son also reported that she had been moving her right arm and chest erratically and screaming during the ambulance ride. Upon their arrival at the ER, the paramedics also offered a brief history. They could not report accurate vital signs from their ride to the hospital, stating that the patient’s blood pressure could not be read due to a device error; her other vital signs had included a respiratory rate of 24, heart rate of 126, and pulse oximetry of 94%. The paramedics had administered oxygen via a nasal cannula and set an intravenous line in the ambulance.

**Figure 1:**
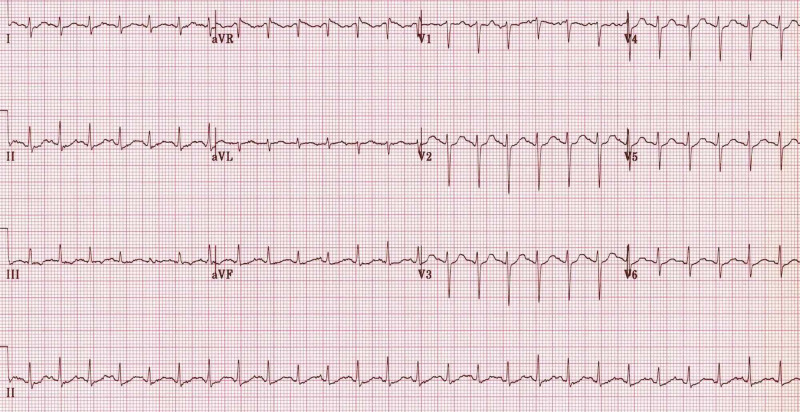
Electrocardiography

After her son and the paramedics supplied their information, the patient was attached to the cardiac monitor, which immediately rendered no signs of cardiac ischemia via electrocardiography (Figure 1). A physical examination indicated an obese, agitated, semicomatose, and gasping condition. She was moving her right arm and holding the right side of her chest intermittently. A cardiovascular examination revealed a regular rhythm and normal heart sounds with no murmur or rubs. Pulmonary, abdominal, and neurological examinations were also normal.

The vital signs recorded were surprising since, while the patient had no fever, her blood pressure was 370/191 mmHg, respiratory rate 22, heart rate 115, and pulse oximetry 95%. Immediate verification of her blood pressure revealed the same reading. A decision to administer 20 mg of intravenous hydralazine and monitor her vital signs under the highest care was made. The first ampule of hydralazine was administered, and after five minutes, the patient’s blood pressure dropped to 330/182 mmHg. The second ampule of hydralazine was then administered, and after another five minutes, her blood pressure dropped to 300/180 mmHg. Finally, the decision to administer 40 mg of hydralazine in two dosages ten minutes apart was made. The patient’s blood pressure dropped to 220/160 mmHg; she began to regain consciousness gradually, with no gasping, and her right arm movement subsided. Her vital signs indicated a heart rate of 118, a respiratory rate of 20, and unchanged pulse oximetry. Another electrocardiograph rendered no ischemia. However, five minutes after the last dose of hydralazine, another ampule was administered in response to unchanged blood pressure since the previous reading; the patient’s blood pressure then dropped to 171/130. She began to feel better and wanted to know what had happened to her. She was alert to time, place, and persons a few minutes later.

**Figure 2:**
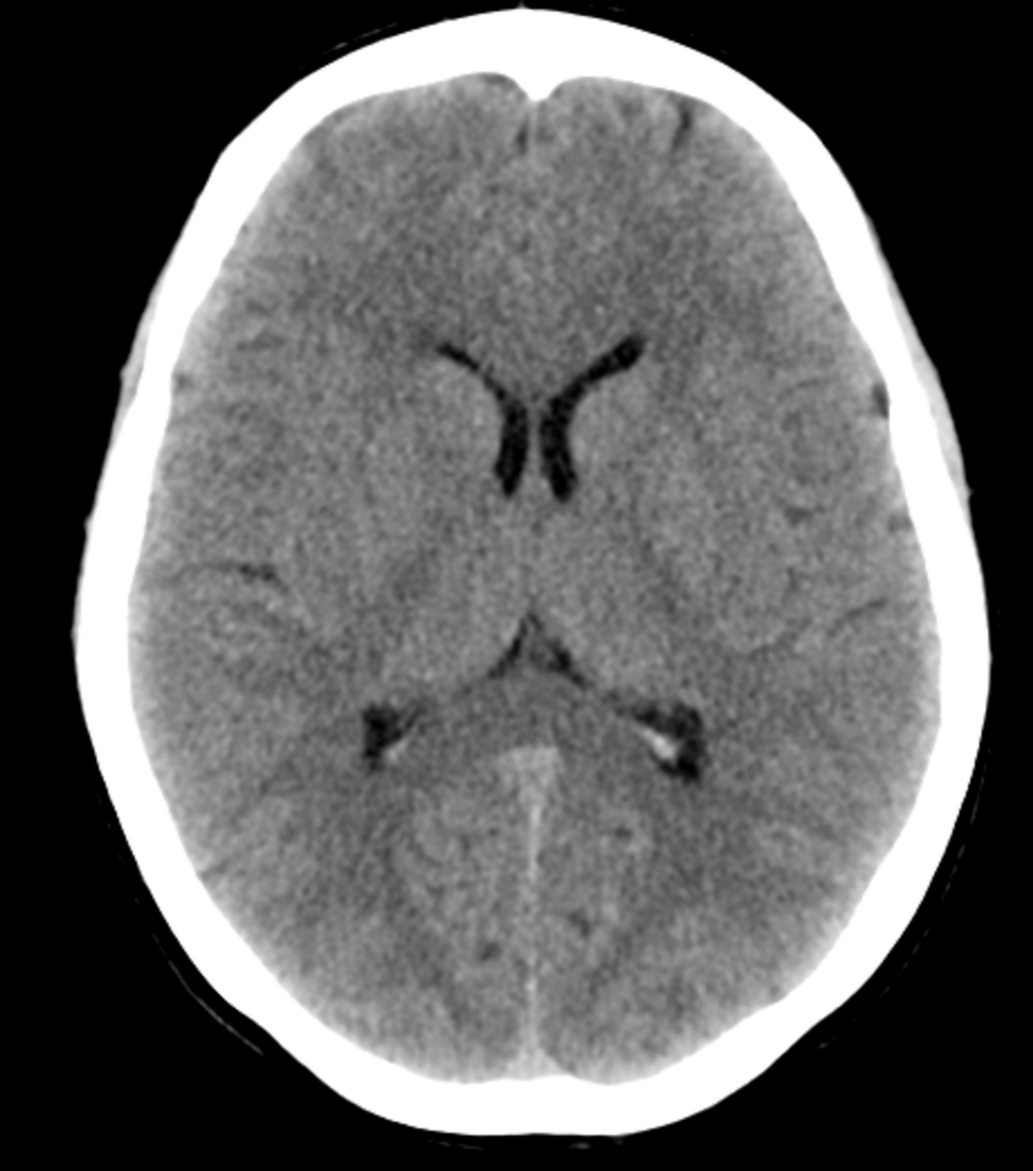
CT scan of the brain

The patient’s current vital signs were as follows: blood pressure of 170/128 mmHg, respiratory rate of 17, heart rate of 119, and pulse oximetry of 94% on room air. She received 50 mg of oral metoprolol and provided a full history. She had been on 10 mg/day of lisinopril for the last five years for hypertension, and she denied having any chronic diseases such as diabetes mellitus, asthma, stroke, angina, and liver or kidney problems. Repeated physical, neurological, cardiac, pulmonary, and gastroenterological examinations were normal. Her blood pressure dropped to 150/100 mmHg 20 minutes after the latest reading. Her vital signs then revealed a heart rate of 105, respiratory rate of 17, and pulse oximetry of 95%. Computer tomography rendered normal findings and no signs of a stroke (Figure 2). Other measurements included 1 mg of creatinine and 25 mg of blood urea nitrogen (BUN). An echocardiograph and abdominal ultrasound were both within the normal parameters. Furthermore, an ophthalmological consultation rendered normal findings.

Before the patient was discharged, her vital signs demonstrated a blood pressure of 130/84 mmHg, a respiratory rate of 16, a heart rate of 79, a pulse oximetry of 97% on room air, and no fever. She was discharged the following day after achieving a stable condition. All laboratory results, such as her complete blood count and comprehensive metabolic panel, included normal limits. She was scheduled for an MRI five days after her discharge, and the results were within normal limits.

## Discussion

Pathophysiology

**Figure 3:**
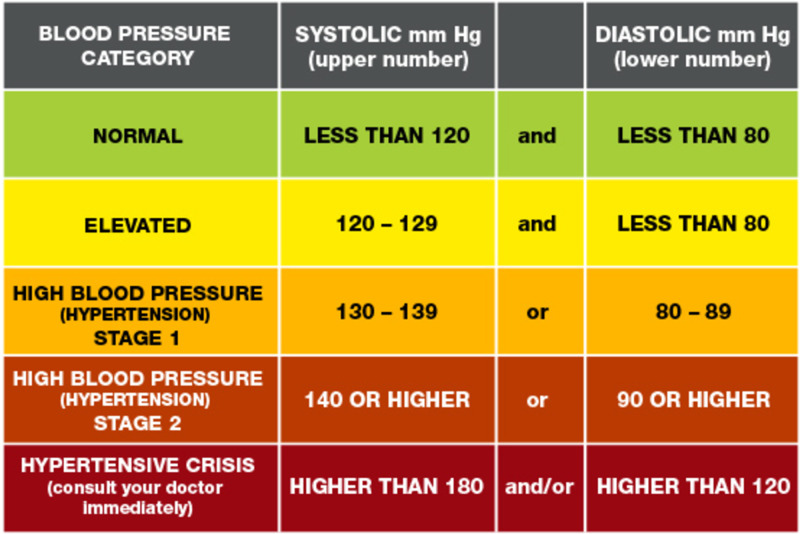
Stages of blood pressure Credit: American Heart Association

Hypertension is not only a common condition but also the leading cause of global death, accounting for 10.4 million deaths annually. This condition affects the high-income more than the low-income population and men more than women. The prevalence of blood pressure increases and adverse impacts on cardiovascular morbidity and mortality are increasing globally regardless of income (Figure 3) [4].

**Figure 4:**
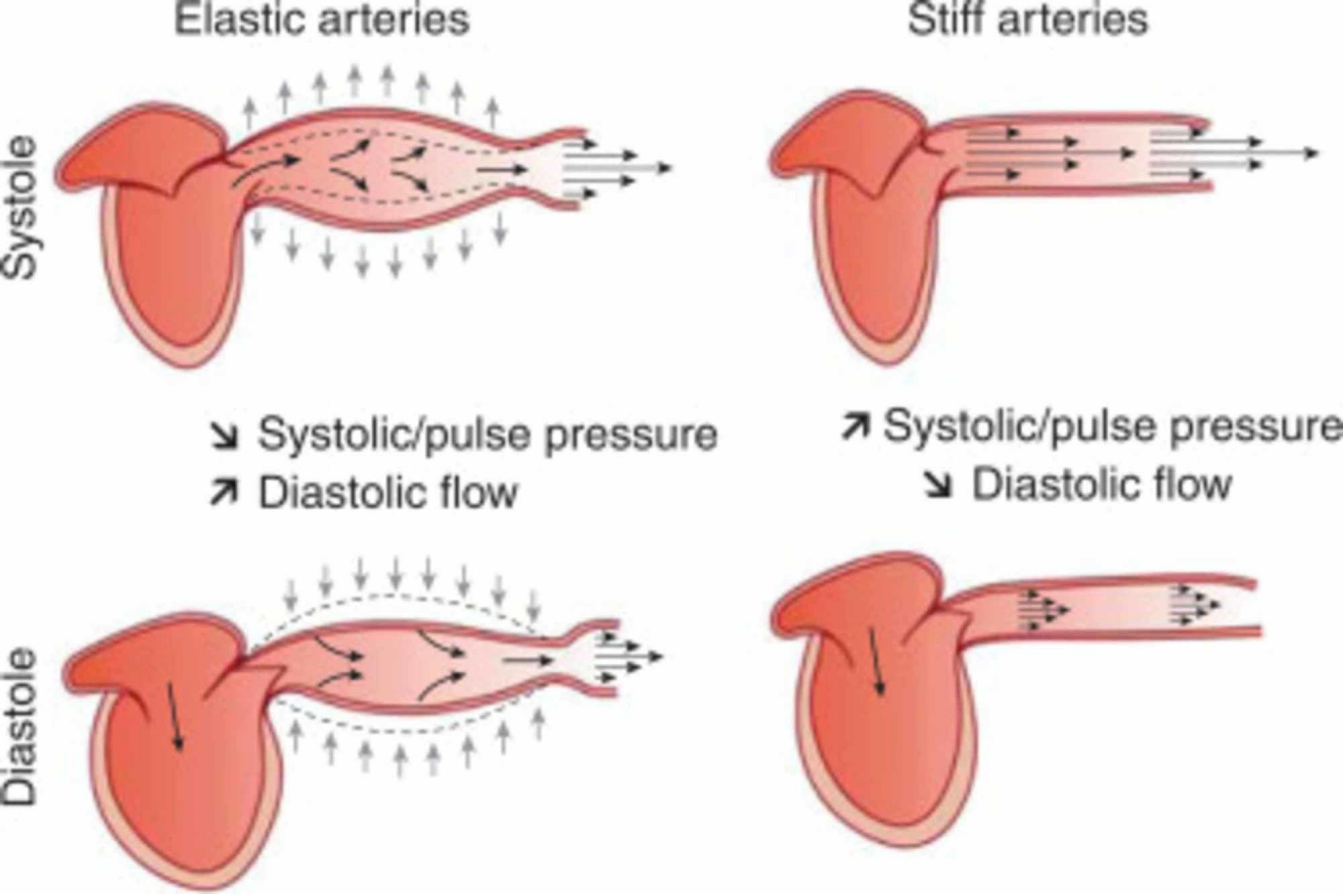
Blood vessel elasticity in response to systolic and diastolic pressure changes Credit: Nature's Real Health

While hypertension is a chronic condition that affects the entire body, the most common organ affected is the heart. Blood pressure is the product of cardiac output and systemic vascular resistance; hypertension may involve an increase in cardiac output, an increase in systemic vascular resistance, or both. Aging individuals have stiffer blood vessels. The loss of elasticity form chronic exposure to pressure from blood passage plays a vital role in their pathophysiology (Figure 4).

**Figure 5:**
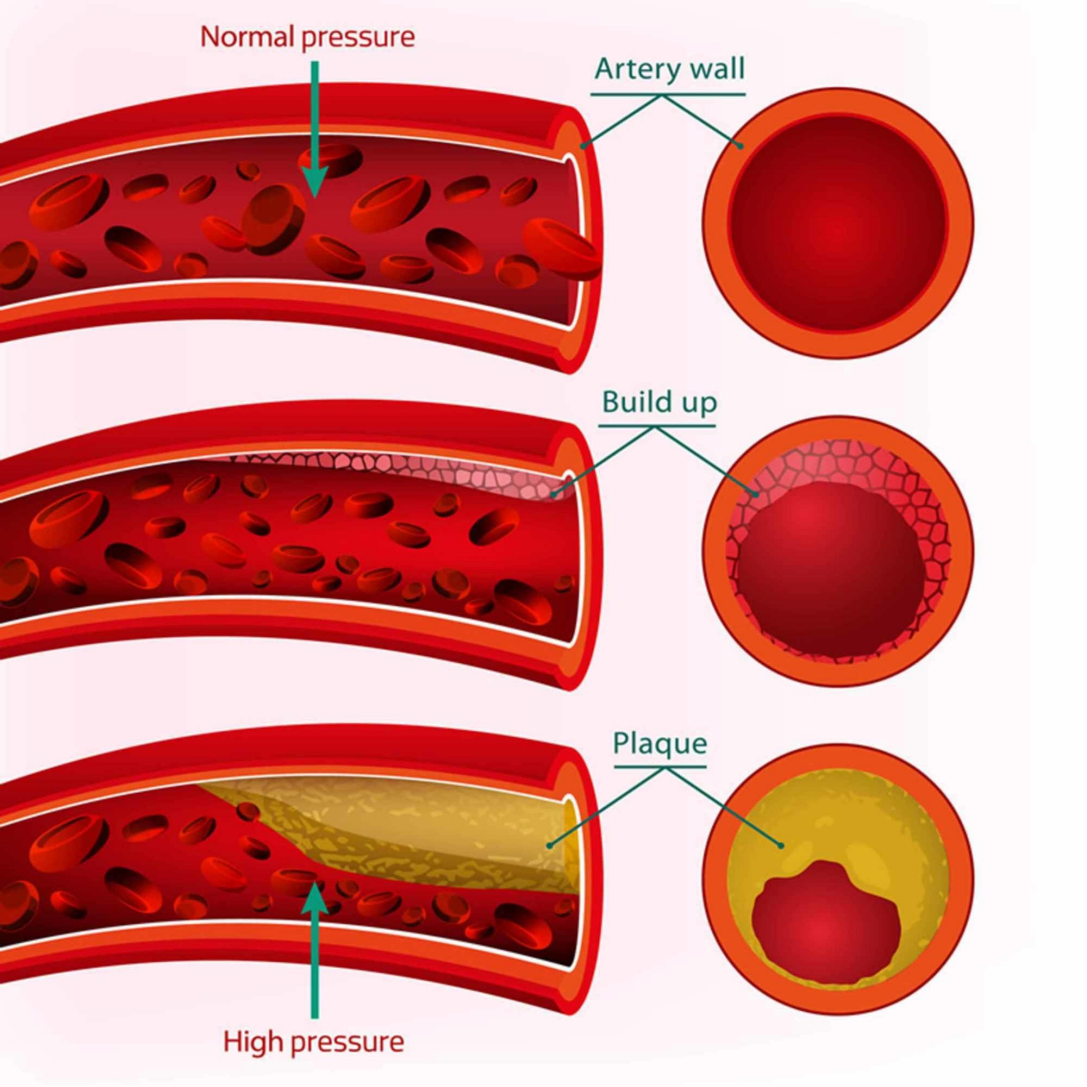
Blood vessel wall in response to high pressure Credit: USA vascular center.

Chronic pressure on the vascular wall leads to an increase in the alpha adrenoreceptor or an increase in the release of peptides such as angiotensin and endothelin, followed by an increase in systolic calcium in the vascular smooth muscle, which causes vasoconstriction. However, angiotensin and endothelin factors lead to an increase in vascular smooth muscle mass in terms of vascular remodeling (Figure 5). Vascular resistance and vascular stiffness produce an imposed load on the left ventricle; left ventricle hypertrophy, coronary arterial disease, and ventricle diastolic dysfunction occur as a result [5].

**Figure 6:**
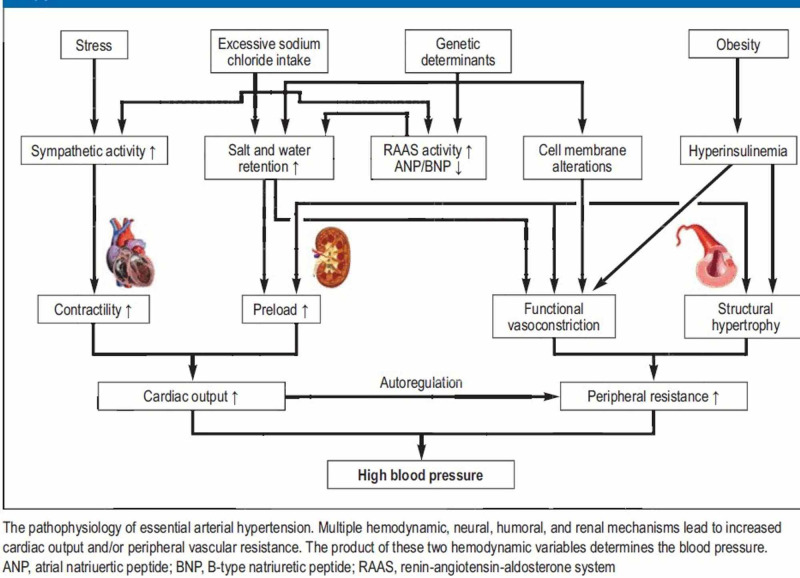
Mechanism of high blood pressure Credit: https://prevent-hypertency.blogspot.com/2012/12/pathophysiology-of-essential.html

Hypertensive patients have an increased release of and sensitivity to norepinephrine, as well as an increased response to stressful stimuli and decreased baroreceptor sensitivity [5]. Two types of hypertension occur: idiopathic hypertension, which includes 90% of hypertension cases, and secondary hypertension, which results from multiple diseases, including renovascular hypertension, acromegaly, obstructive sleep apnea, adrenal tumors, and congenital adrenal hyperplasia; using drugs such as cocaine, amphetamine, oral contraceptive pills, and decongestants; and herbal therapy (Figure 6) [6].

The regulation of blood pressure is a complex mechanism. Neurogenic control including the dorsal medulla (nucleus solitarius), pons, and midbrain, decreases the efferent sympathetic activity, which finally leads to bradycardia and vasodilation when the arterial baroreceptors respond to vessel wall distention by increasing the afferent impulse activity.

A hypertensive emergency is a true medical emergency that requires prompt treatment to reduce blood pressure and prevent organ damage. The pathophysiology is not well understood; nevertheless, the failure of normal autoregulation and an abrupt rise in systemic vascular resistance are often the first steps in the disease process. An increase in systemic vascular resistance occurs with the release of vasoconstrictors from the vessel wall. Incremental pressure changes in the vessel lead to endothelial damage. Local intravascular activation of coagulation cascade releases more vasoconstrictors, and fibrinoid necrosis of small blood vessels. If this process is not prevented as soon as possible, further vascular injury, tissue ischemia, and autoregulation dysfunction occur [7].

The risk factors for hypertension include age (the most important one), race (African Americans are more vulnerable), family history, obesity, alcohol, diet, a sedentary lifestyle, and stress [6]. While the causes of hypertensive emergency are extensive, the most important ones are medication noncompliance, stroke, eclampsia, adrenal tumor, angina, medication interaction, and stress [8].

**Figure 7:**
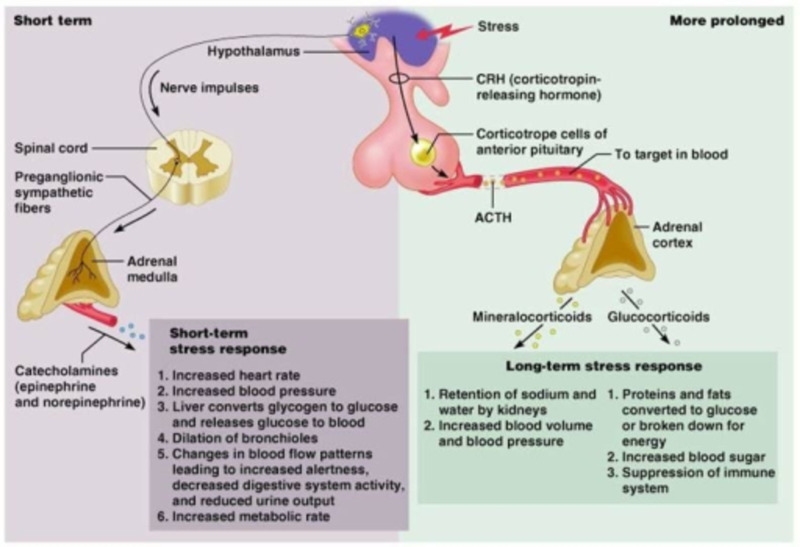
Physiological changes in response to stress Credit: www.studyblue.com

Stress is now a major predicament, whether it is related to environmental or psychological factors, as both can trigger a hormonal change in the body. The initial response to stress begins in the amygdala, which receives input from various brain centers, including visual and auditory systems. In other words, when an individual experiences a critical situation, data is transmitted to the amygdala, which interprets that information and sends it to the hypothalamus; the hypothalamus then communicates with the rest of the body via the autonomic nervous system to control vital signs such as breathing, blood pressure, heart rate, and the dilation or constriction of blood vessels. The autonomic nervous system is divided into sympathetic (fight-or-flight response) and parasympathetic (rest or digest) systems. The activation of the sympathetic nervous system during emotional stress ultimately sends an impulse to the adrenal medulla to release catecholamines, including epinephrine and norepinephrine, which both lead to an increase in vital signs such as elevated blood pressure, tachypnea, and tachycardia. If the stressors persist, the surge in catecholamines also persists, resulting in more damage to the vascular endothelium and worsening the condition [9]. These catecholamines usually affect alpha and beta receptors. Alpha receptor activation in the blood vessels leads to vasoconstriction and elevated blood pressure, whereas beta receptor activation in the heart or other organs leads to variable changes, such as the contraction or relaxation of smooth muscles and an increased heart rate (Figure 7) [10].

The first organ that a hypertensive emergency affects is the kidney, which suffers from ischemia and activates the renin-angiotensin-aldosterone system (RAAS). The condition therefore worsens due to the further elevation of blood pressure. In addition to the activation of the RAAS, other factors such as endothelin, vasopressin, and catecholamines play a vital role in the pathogenesis of hypertensive emergency [11].

The goal is to find any organ damage (including angina, myocardial infarction, stroke, acute kidney injury, hypertensive encephalopathy, eclampsia, preeclampsia, left ventricular failure, pulmonary edema, aortic dissection, and retinopathy) and attempt to prevent the progression as fast as possible. The patient could present with shortness of breath, chest pain, confusion, coma, vomiting, or vision changes. A physical examination should initially focus on any cardiac symptoms, such as gallops, murmurs, and S3 sounds; a lung examination on rales; and a neurological examination on seizures. Moreover, a mental status funduscopic examination should exclude papilledema and other conditions. Monitoring the patient’s vital signs is an important step. Indeed, the patient should be managed in the ICU under high care and supervision [12,13].

**Figure 8:**
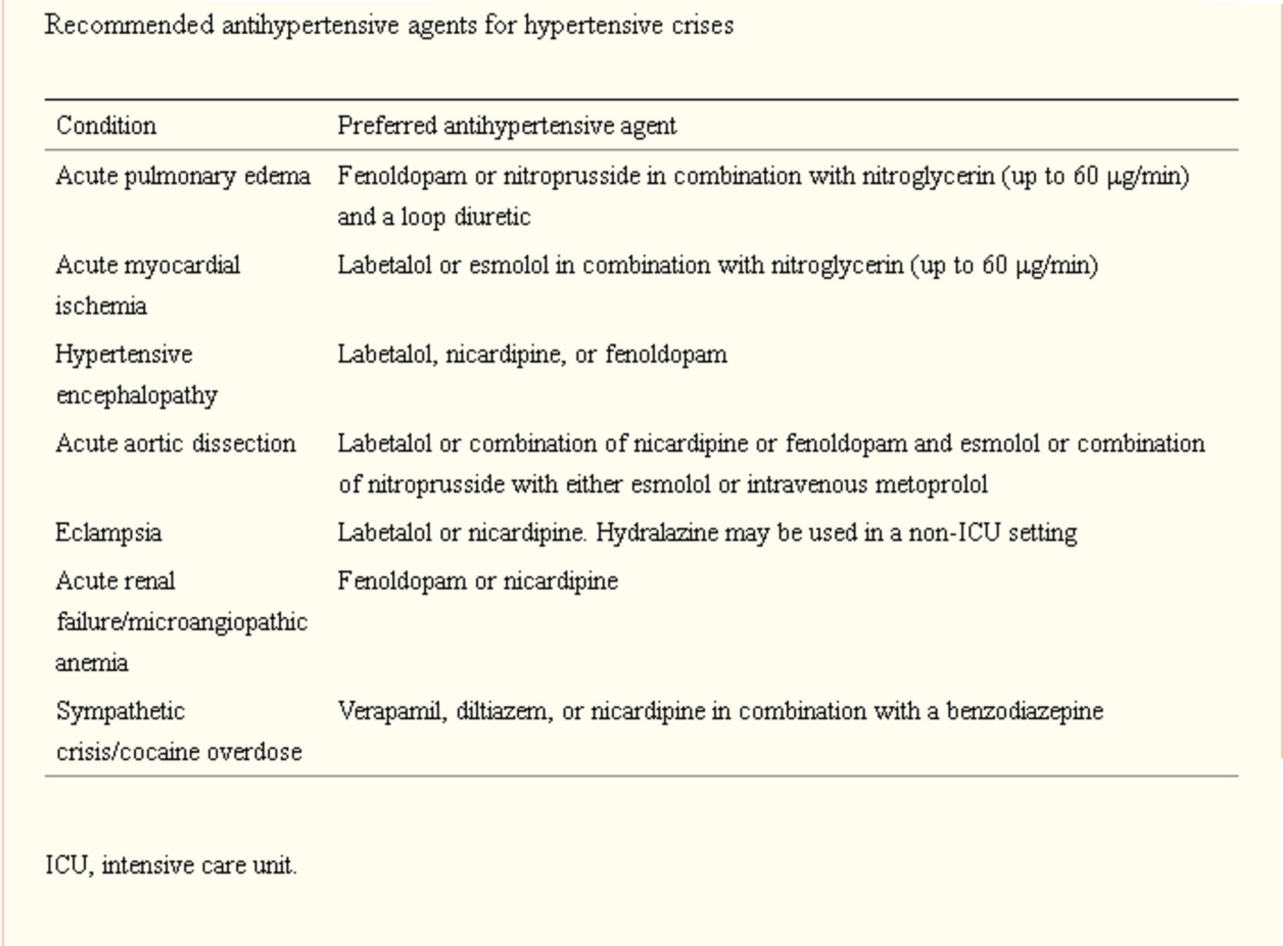
Antihypertensive medications Credit: Merck Manuals [13]

First, lowering the patient’s blood pressure should be done within minutes by lowering the mean arterial pressure by up to 25% in the first thirty minutes and if the patient remains stable then further lowering can be done over the next hour. Attaching the patient to a cardiac monitor is a crucial step in blood pressure management [13]. Once the patient’s blood pressure has been lowered to a safe level, investigations into his or her full history and a physical examination should be conducted. The laboratory investigations include a complete blood count (CBC); a comprehensive metabolic panel (CMP); electrocardiography; echocardiography; brain, chest, and abdomen images, including CT scans or MRI; a drug panel; a thyroid-stimulating hormone (TSH) test; a catecholamine blood test; and a growth hormone test [14]. Various antihypertensive medications are available for use in hypertensive crises (Figure 8) [13].

## Conclusions

A hypertensive emergency usually presents with elevated blood pressure and end-organ damage, including heart, eye, kidney, and brain damage. Remarkably, the current case presented with the usual elevated blood pressure but without end-organ damage, which makes it a unique case to report. The patient was successfully treated with hydralazine and suffered no complications, including stroke and myocardial infarction.
